# Genetic predisposition to prostate cancer: an update

**DOI:** 10.1007/s10689-021-00227-3

**Published:** 2021-01-24

**Authors:** Holly Ni Raghallaigh, Rosalind Eeles

**Affiliations:** Oncogenetics Team, Division of Genetics & Epidemiology, The Institute of Cancer Research, Sir Richard Doll Building, 15 Cotswold road, Sutton, SM2 5NG UK

**Keywords:** Prostate cancer, Germline genetics, Prostate cancer risk, Familial prostate cancer, Hereditary prostate cancer

## Abstract

Improvements in DNA sequencing technology and discoveries made by large scale genome-wide association studies have led to enormous insight into the role of genetic variation in prostate cancer risk. High-risk prostate cancer risk predisposition genes exist in addition to common germline variants conferring low-moderate risk, which together account for over a third of familial prostate cancer risk. Identifying men with additional risk factors such as genetic variants or a positive family history is of clinical importance, as men with such risk factors have a higher incidence of prostate cancer with some evidence to suggest diagnosis at a younger age and poorer outcomes. The medical community remains in disagreement on the benefits of a population prostate cancer screening programme reliant on PSA testing. A reduction in mortality has been demonstrated in many studies, but at the cost of significant amounts of overdiagnosis and overtreatment. Developing targeted screening strategies for high-risk men is currently the subject of investigation in a number of prospective studies. At present, approximately 38% of the familial risk of PrCa can be explained based on published SNPs, with men in the top 1% of the risk profile having a 5.71-fold increase in risk of developing cancer compared with controls. With approximately 170 prostate cancer susceptibility loci now identified in European populations, there is scope to explore the clinical utility of genetic testing and genetic-risk scores in prostate cancer screening and risk stratification, with such data in non-European populations eagerly awaited. This review will focus on both the rare and common germline genetic variation involved in hereditary and familial prostate cancer, and discuss ongoing research in exploring the role of targeted screening in this high-risk group of men.

## Background

Prostate cancer (PrCa) is the second most common malignancy affecting men worldwide, and the most common cause of cancer-related death in men in the United Kingdom, with a significant associated health burden due to its incidence. Most men with PrCa will have unaffected overall survival due to the biologically ‘indolent’ nature of the majority of disease, even if treatment is required. Much controversy exists in the role of PrCa screening, as PSA has a propensity to detect a large amount of cancers ultimately destined to be clinically insignificant, and is poor at discriminating between such men and those harbouring lethal disease who would benefit the most from identification and treatment.

**Fig. 1:**
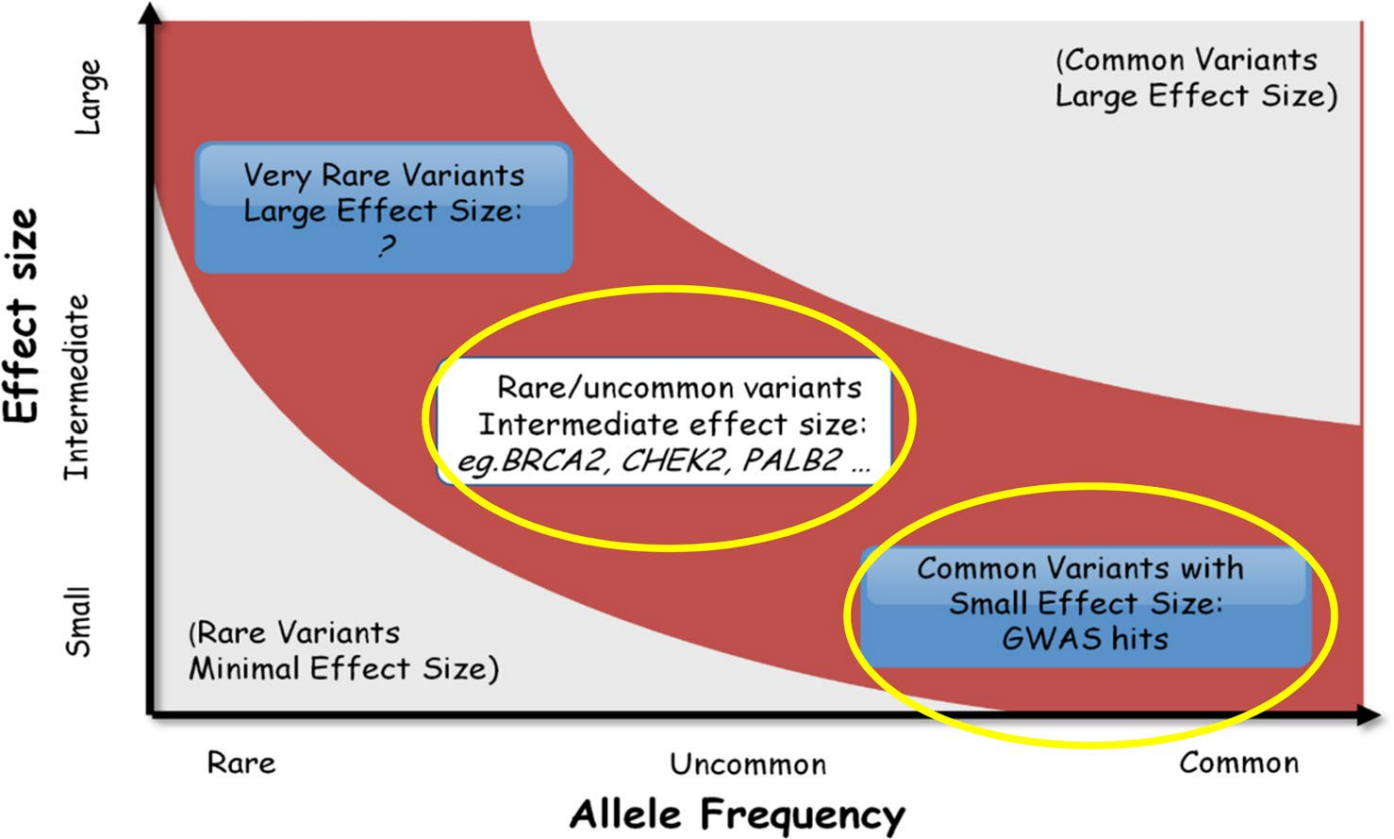
Reproduced and adapted from Maniolo et al. Diagram showing the spectrum of genetic variants in polygenic disease i.e. PrCa. The X-axis plots the risk allele frequency and effect size along the y-axis. The top right corner represents common variants with large effect sizes (none known). The bottom left corner represents rare variants with small effect size. Such variants would be of limited clinical interest. Candidate gene and linkage analyses have discovered rare variants (i.e. *BRCA1/2, HOXB13* which produce moderate effect sizes. Genome wide association studies (GWAS) have discovered common variants conferring small to modest effect sizes. Those variants circled in yellow represent the germline genetic variations we incorporate into PRS; (common variants) and panel testing (eg. *BRCA2*) [4]. (Reprinted by permission from Springer Nature: Nature**.** Finding the missing heritability of complex diseases, Maniolo et al. ©2009)

Not all men are at equal risk for developing PrCa, which is a polygenic disease with a large amount of heritability. Men with a brother or father affected with PrCa have at least a two-fold risk of developing PrCa compared to men without a family history (FH), with the risk increasing further if the affected first degree relative (FDR) had early onset disease (≤ 55 years) with a relative risk (RR) of 3–5 [1]. Both high-risk monogenic and polygenic causes for PrCa exist, together explaining approximately 45% of familial disease [2, 3] (Fig. 1). The potential for ‘clustering’ of PSA screening to occur in men with affected relatives has been discussed as a mechanism for the increasing the numbers of screen-detected PrCas in men with a FH of the disease, thereby contributing to the increased incidence of PrCa in men with affected relatives versus families in whom multiple men are diagnosed by clinical symptoms.

### Hereditary prostate cancer (HPC)

**Fig. 2:**
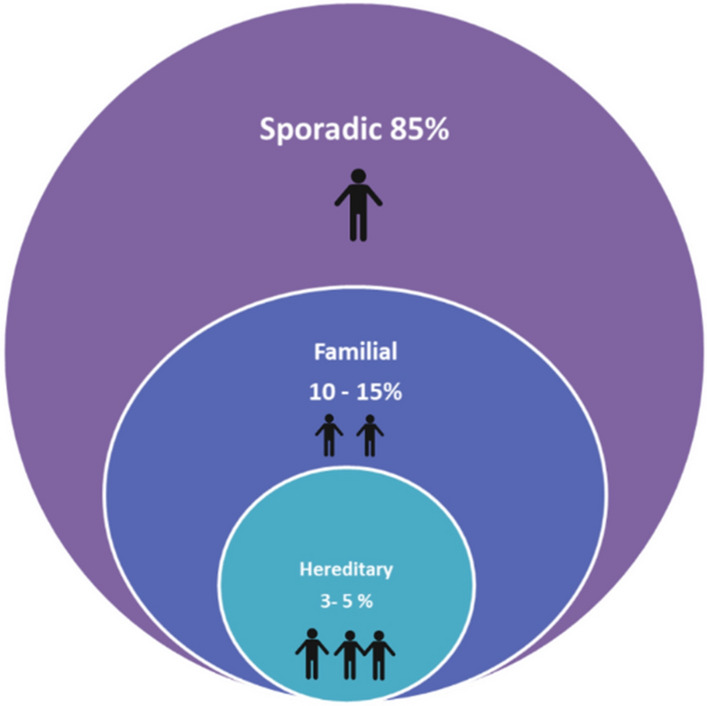
Reproduced and modified from Klein et al. Schematic representation of the proportion of PrCa caused by HPC and familial PrCa [9]. Reprinted with permission from Springer Nature: Prostate Cancer and Prostatic Diseases. Does a family history of prostate cancer result in more aggressive disease? Klein et al. ©1999

This is a specifically defined scenario based on a man’s pedigree, with three categories: (1) PrCa in three generations, (2) two cases of PrCa with an age of onset < 55 years or (3) three first-degree relatives with the disease. It is still unclear if the biology of PrCa in men with HPC is more aggressive or different to those with ‘sporadic’ PrCa, but men with HPC do tend to have earlier onset disease. This specific subtype of familial PrCa was described by Carter et al. in 1993, and accounts for approximately 3–5% of all prostate cancers [5] (Fig. 2) following segregation analyses and studies performed in twins and the Utah population database. In men with PrCa diagnosed at ≤ 55 years, it is found in up to 43% of cases [6, 7]. It is worth noting that mendelian inheritance pattern of HPC has primarily only been studied in Caucasian populations.

### Familial prostate cancer

This describes the remainder of men with a ‘FH’ of PrCa (who do not fulfil the above criteria). Men with familial PrCa still have a significantly higher lifetime risk of developing the disease, with a two–eightfold increase reported [8] and worsening risk with the number of relatives affected. Familial PrCa is likely caused by a combination of dominant, moderate/high-risk genes, risk modulating-genes, common low-moderate risk variants, environmental exposures and advancing age.

#### Twin studies

Scandinavian twin studies have described the large effect of the heritability in PrCa in a study of over 44,000 pairs of both monozygotic (identical) and dizygotic (non-identical) twins. Lichenstein et al. demonstrated concordance between identical and non-identical twins i.e. the concordance for identical twins was 0.21 and 0.06 for non-identical twins meaning a man with an identical twin affected with PrCa has a 21% probability of having PrCa himself (6% for non-identical twins). They also showed a higher absolute risk (up to age 75) of PrCa in men with an affected identical twin (18%) compared to those with a non-identical twin (3%) and showed the difference in age of onset of PrCa was smaller in concordant pairs of identical twins (5.7 years; SD 3.39) with PrCa than in concordant pairs of non-identical twins (8.8 years; SD 5.66). They estimated that 42% of PrCa risk in these (Swedish, Finnish and Danish) men was due to heritable factors (95% CI 0.29–0.50) [10]. Hjelmborg et al. estimated the cumulative incidence of PrCa to provide detailed estimates of familial risk amongst identical and non-identical twins in the NorTwinCan collaboration, comprising four twin cohorts from Norway, Sweden, Denmark and Sweden (143, 467 men). At all ages, the risk of PrCa in both identical and non-identical twins was higher than the overall population incidence with the risk for those who had an identical twin already diagnosed with PrCa three-fold higher than the corresponding risk for non-identical twins. Among twin pairs where both twins had PrCa, there was a significantly shorter time between the diagnosis in the first and second twin among the identical compared to the the non-identical pairs. The mean difference was 4.6 years (SE, 0.43) and 7.8 years (SE, 0.45) respectively [11].

A Swedish study reporting from a family-database of over 9 million participants reported a PrCa standardized incidence ratio (SIR) of 23.72 for men whose father and brother were affected [12]. Another group screened 34 first-degree relatives (sons/brothers) of 17 sets of (two) brothers with PrCa, using a combination of PSA, digital rectal examination (DRE) and trans-rectal ultrasound guided (TRUS) biopsy. Clinically significant, asymptomatic PrCa was found in 8 (24%) men with a reported RR of developing PrCa of 5–11 [13].

### Is the phenotype different?

Evidence for differing disease biology and trajectories between sporadic, familial and hereditary PrCa is varied. Early work by Kupelian et al. showed poorer biochemical recurrence (BCR) rates at 5-years following radical prostatectomy in men with familial PrCa (one FDR affected with PrCa) compared with those without (n = 529 with 12% of the cohort having a positive FH). FH remained an independent predictor of BCR after adjusting for age, histology, stage and surgical pathology variables [14, 15]. However in a similar analysis of 708 men undergoing radical prostatectomy by Bova et al. with longer follow-up, no differences in BCR were seen between men with familial PrCa or HPC compared with men with sporadic PrCa when cases were disease and age-matched [16]. A recent retrospective analysis of 9,459 PrCa patients from an Australian cancer outcomes database reported on the effect of FH on overall survival (OS) after adjustment for age, NCCN risk category and year of treatment. They found better overall survival (OS) outcomes in men with a FH compared to those without (HR 0.74, 95% CI 0.57–0.97, p = 0.027) with no difference in outcomes between men undergoing radical prostatectomy or radiotherapy, or PrCa specific-mortality (HR 0.74, 95% CI 0.50–1.10) [17].

With regards to clinical features including age at onset, histology and presenting PSA, Gronberg retrospectively analysed 74 families with familial and HPC compared with men without any FH. They showed that men with likely HPC harboured aggressive histology at diagnosis, had an earlier age of onset by 2 years and had worse stage at diagnosis than men with unlikely HPC and men with no FH [18]. In an analysis of 481,000 men in the Cancer Prevention Study II (CPS-II), 3% of men reported a FH of PrCa in one FDR and 0.05% reported a history in two FDRs. Men who had any FH of PrCa had a 60% increase in risk of having fatal PrCa compared to those without, with a greater magnitude of effect if their affected relative was diagnosed before age 65 (RR of fatal PrCa 2.03; 95% CI 1.33–3.09) [19]. Elshafei et al. assessed the risk of FH on having a positive prostate biopsy in men with a clinical suspicion of PrCa due to raised PSA or abnormal DRE in a single centre from 2000 to 2010. They found a significant association between FH status and the presence of both low grade and high grade cancer on initial biopsy. In all men with a positive biopsy, men who had a FH of PrCa were younger and had a lower PSA than men without a FH. In multivariable analysis of men with a FH, prostate volume and PSA were significantly associated with high-grade disease [20].

Brandt et al. reported an increased risk of fatal PrCa in men whose father or brother had died from PrCa in an analysis of the Swedish Family Cancer database. They demonstrated a standardised mortality ratio of death from PrCa in men with a father (2.04) or a brother (2.75), with a risk of incident PrCa of 2.28 in men whose father died from PrCa and 3.25 in men whose brother died from PrCa [21].

In summary, evidence for an earlier age at onset in men with familial PrCa exists but convincing evidence for a difference in the clinical course or pathological characteristics is lacking. There is good evidence however for a significant difference in disease aggressiveness and disease-specific survival in men with a known pathogenic germline variant in a DNA repair gene, as discussed below.

## Specific germline genetic variants involved in PrCa

Variants in genes involved in DNA mismatch repair, particularly *BRCA1/2*, *ATM, CHEK2,* and *NBN* have been associated with an increased risk of developing PrCa in men with advanced/metastatic PrCa unselected for FH as well as in men with familial PrCa.

A review by Pritchard et al. of 692 men with mCRPC revealed a germline DNA repair-gene variant in 11.8% of all men, across 16 genes including *BRCA1/2, ATM, CHEK2, PALB2* and *RAD51D* [22]. These men were unselected for age at diagnosis or FH status. In men with localised PrCa, a lower frequency of germline DNA repair gene variant of 4.6% was found (however when specifically grouping men by NCCN risk criteria, 2% of men with low-intermediate risk had germline variants in DNA repair genes). Men who carry germline variants in *BRCA2* with metastatic disease have been shown to have superior responses to PARP inhibition and platinum chemotherapy, signalling the emerging importance of knowing a patient’s variant status, especially if presenting with advanced or metastatic disease [23–25].

Nicolosi et al. performed a cross-sectional study of 3607 men with PrCa, unselected for FH, age or disease stage referred to clinical genetics for germline testing between 2013 and 2018. They found 17.2% of men carried pathogenic germline variants, of which 30.7% were *BRCA1/2* variants, 4.5% were due to *HOXB13*, 14.1% *CHEK2* and 9.6% due to *ATM* [26].

**Fig. 3:**
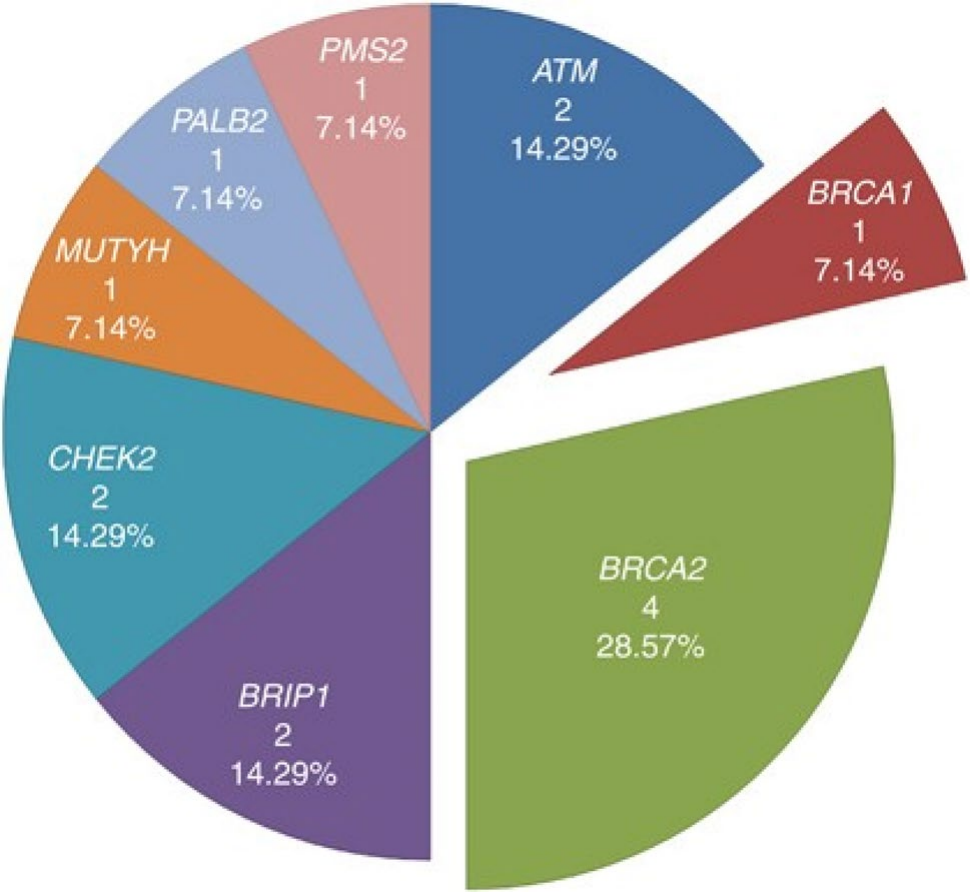
Reproduced from Leongamornlert et al. Distribution of pathogenic germline variants in 191 men with at least  ≥ 3 cases of PrCa in their family [27]. Reprinted by permission from Springer Nature: on behalf of Cancer Research UK: Springe Nature. Br J C. Frequent germline deleterious variants in DNA repair genes in familial prostate cancer cases are associated with advanced disease, Leongamornlert et al. ©(2014)

In an analysis of a European cohort of men with a FH of PrCa in the United Kingdom Genetics Prostate Cancer Study (UKGPCS), 7.3% of PrCa patients with a positive FH (of three cases of PrCa) were found to carry a pathogenic germline variant. The most frequent variant was in *BRCA2* (28.57% of all variants), and importantly there was a significant association between genetic variant carrier status and nodal and metastatic disease (Fig. 3). [27]

### NBN

Cybulski et al. genotyped over 3750 Polish men with PrCa for variants in *BRCA1*, *CHEK2* and *NBN*.

A founder pathogenic variant (675del5) in *NBN* is found in approx. 1 in 750 of the Polish population, conferring a three-fold increase in risk of PrCa and an apparent significant effect on overall survival after adjusting for age, stage and tumour grade. *CHEK2* variants did not appear to have a similar effect on survival but were found more commonly in men with familial PrCa, and were more common than *BRCA1* variants (Table 1). It is estimated that variants in *NBN* and *CHEK2* account for 1.4% and 5% of all prostate cancers in Poland respectively [28]. In a recent analysis of the contribution of *NBN* founder alleles to PrCa specific survival and risk, the 657del5 variant was associated with significantly worse survival (p = 0.001, HR 1.6; 95% CI 1.1–2.5) [29]

**Table 1:** Reproduced and adapted from Cybulski et al. Frequency of germline variants of *BRCA1, CHEK2* and *NBN* in controls, familial cases and cases unselected for FH *Mut* variant [28]

	Controls (*n* = 3956) No. (%)	Unselected cases (*n* = 3750) No. (%)	OR	95% CI	*p*-Value	Familial cases (*n* = 412) No. (%)	OR	95% CI	p Value
Any *BRCA1* mut	17 (0.4%)	14 (0.4%)	0.9	0.4–1.8	0.8	4 (1.0%)	2.3	0.8–6.8	0.3
*NBN *657del5	23 (0.6%)	53 (1.4%)	2.5	1.5–4.0	0.0003	10 (2.4%)	4.3	2.0–9.0	0.0001
*Any CHEK2 mut*	228 (5.8%)	383 (10.2%)	1.9	1.6–2.2	< 0.0001	59 (14.3%)	2.7	2.0–3.7	< 0.0001

Reprinted by permission from Springer Nature: on behalf of Cancer Research UK: Br J C. An inherited NBN variant is associated with poor prognosis prostate cancer, Cybukski et al. © (2012)

### CHEK2

*CHEK2* variants have been implicated in familial and hereditary PrCa, and are also known to occur in breast cancer [30]. Pathogenic variants of *CHEK2* are rare in men of Asian, Hispanic or African ancestry. Seppala et al. genotyped 537 men with PrCa unselected for FH, 120 men with HPC and 480 healthy controls for the truncating 1100delC and missense I157T *CHEK2* variants. Both variants were significantly associated with PrCa in men with HPC [31]. A pooled OR of developing PrCa in those with a *CHEK2* 1100delC variant of 1.98 (95% CI 1.23–3.18) and 3.39 (1.78–6.47) has been found for unselected and familial cases respectively [32]. The I157T variant occurs more frequently in Finish and Polish populations and was found in 16% of familial cases of PrCa (OR 3.38, 95% CI 2.0–7.4; p = 0.00002) vs 7.8% of unselected cases (OR 1.7, 95% CI 1.05–2.7, p = 0.03) and 4.8% of controls [33]. The I157T variant has also been described as occurring more commonly in breast cancer cases in German and Belarussian populations [34]. The 1100delC variant is more common in Northern Europe.

### HOXB13

Karlsson et al. genotyped two population-based Swedish case–control samples; CAPS and Stickhokm-1. Carriers of a rare missense variant (*G84E*) of the *HOXB13* gene have a 33% risk of developing PrCa (95% CI 23–46), compared with a 12% risk in non-carriers (95% CI 11–13) when studied in a Swedish population. This variant was present in 1.3% of population controls and > 4% of cases (CAPS: OR 3.4; 95% CI2.2–5.4; Stockholm-1: OR 3.5; 95% CI 2.4–5.2) [35]. Further large-scale analysis of 4,000 prostate cancer case in Finland for this specific variant revealed a significantly higher carrier rate amongst (unselected) men with PrCa (3.5%) and those with a FH (8.4%) compared to controls (OR 8.8; 95% CI 4.9–15.7) [36]. In a separate study of 5,083 unrelated European subjects who had PrCa, Ewing et al. found the carrier rate of the (*G84E*) variant was increased by a factor of approximately 20 compared with 1401 controls (OR 20.1; 95% CI 3.5–8.3.3). This variant was significantly more common in men with disease at a young age (< 55 years) and with a positive FH (carrier frequency 3.1%; OR 5.1, 95% CI 2.4–12.2), than those without a FH and diagnosed > 55 years (carrier frequency 0.6%) [37]. This pathogenic variant therefore seems particularly significant in young men with PrCa and with a strong FH in Finnish and Swedish populations.

Recently, Nyberg et al. predicted age-specific cumulative risks for carriers of the G84E *HOXB13* variant for developing PrCa under varying pedigrees of FH. The average predicted PrCa risk by age 85 was 62% compared with 15% for non-carriers. For a G84E variant carrier with an affected father, the risk estimate ranged from 69 to 92% depending on the father's age at PrCa diagnosis, and for a man with two affected FDRs, the risk estimate ranged from 70 to 98%. A higher RR (5.96) was also noted for men in more recent birth cohorts (95% CI 4.01 - 8.88) [38].

### BRCA1/2

Variants in *BRCA1/2* are rare with an estimated population prevalence of 0.2–0.3%. The Ashkenazi Jewish population is enriched for variants in these genes with a frequency of approximately 2–2.5% of individuals carrying a variant in *BRCA1/2* (12% of those with a history of female breast cancer and 17% of those with ovarian cancer) and 3.2–4% of men with PrCa [39].

Germline deleterious variants in *BRCA1/2* genes increase the risk of developing PrCa, with variants of both genes reported to increase the risk of PrCa in male carriers by three and seven-fold respectively [40–43]. Male relatives in breast cancer families have a 2–threefold risk of PrCa [44]. It has been suggested that the risk for male *BRCA1* pathogenic variant carriers is lower than previous estimates and that *BRCA2* variant carriers have a significantly higher RR of up to 23-fold at age 60 [45, 46]. Furthermore, *BRCA2* variants may not only be involved in susceptibility to PrCa, but also disease aggressiveness [44], with specific *BRCA2* sequence variants demonstrating an elevated risk [47]

No studies to date have investigated whether there is an optimal treatment strategy specifically for *BRCA1/2* pathogenic variant carriers who develop PrCa. An Icelandic study showed a mean survival of only 2.1 years in men with PrCa with the (founder) 999del5 *BRCA2* variant compared with non-carriers after adjustment for stage and grade [48]. Two further retrospective analyses found an association between *BRCA1/2* status and higher risk of unfavourable histology, disease recurrence and cancer specific-survival (CSS) with a difference of 8.6 years versus 15.7 years between *BRCA1/2* pathogenic variant carriers and non-carriers [49, 50]. Castro and colleagues also showed poorer outcomes (3,5 and 10-year CSS) in men with *BRCA1/2* variants undergoing radical treatment (surgery/radiotherapy) for PrCa when compared with non-carriers [51]. The PROREPAIR-B study reported shorter time to receiving androgen deprivation therapy (ADT) and a reduced median CSS in men with *BRCA2* variants and demonstrated *BRCA2* status as an independent prognostic factor affecting survival in men with metastatic castrate-resistant PrCa [52].

**Fig. 4:**
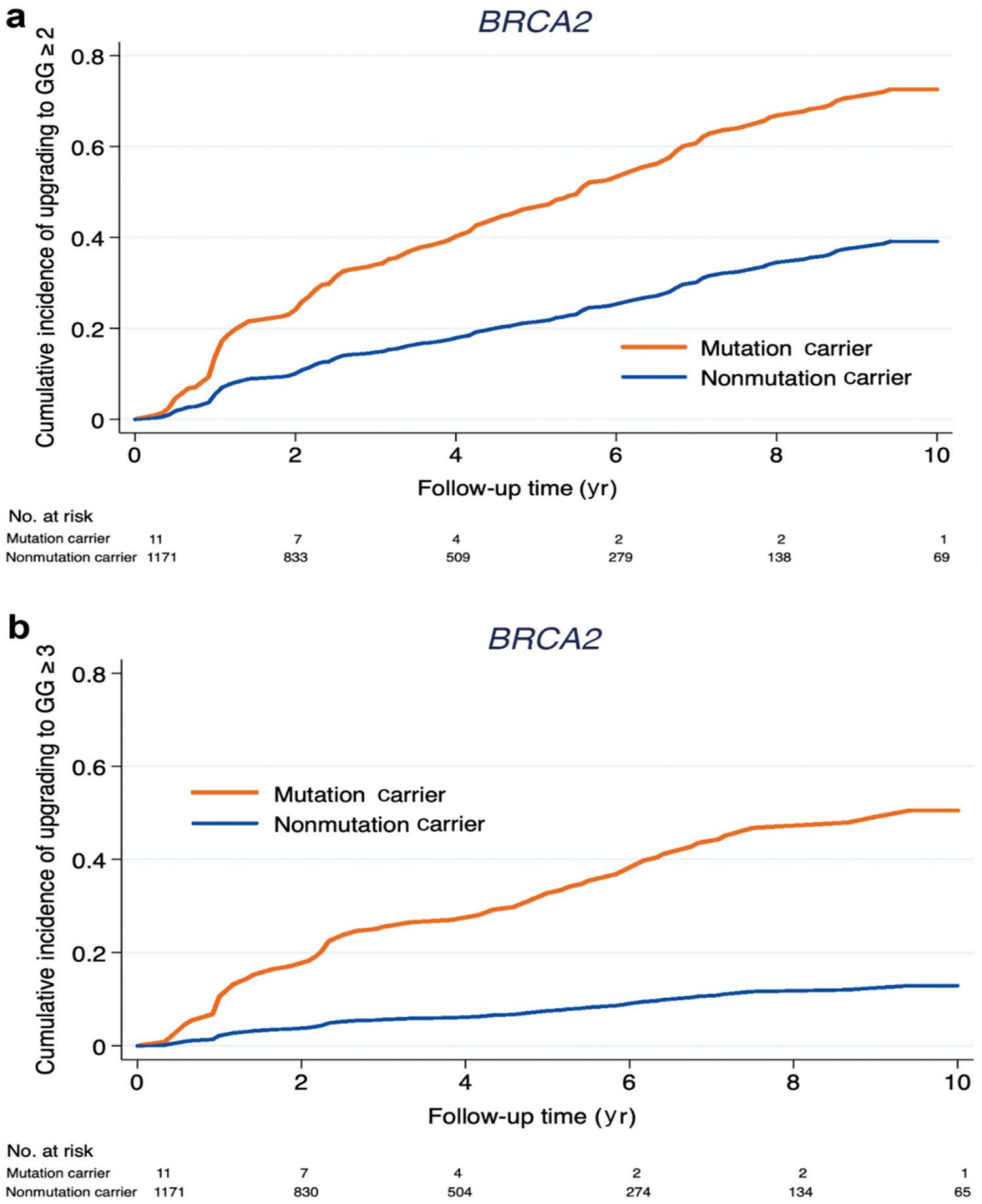
Reproduced from Carter et al. Risk of disease upgrading after diagnostic biopsy among carriers and noncarriers of variants in *BRCA2* only who were initially diagnosed with GGG 1 (Gleason score 3 + 3) : **a** upgrading after diagnostic biopsy to GGG 2 or above (Gleason score 3 + 4 or above); **b** upgrading after diagnostic biopsy to GGG 3 or above (Gleason score 4 + 3 or above) [53]. Reprinted from European Urology, 75(5): Carter et al. Germline variants in ATM and BRCA1/2 are Associated with Grade Reclassification in Men on Active Surveillance for Prostate Cancer, p743-49 ©2019, with permission from Elsevier

As described, men harbouring pathogenic variants in *BRCA1/2* and *ATM* have a worse clinical phenotype. Men are increasingly choosing Active Surveillance (AS) as a treatment option for localised PrCa of favourable risk, due to the avoidance of the morbidity associated with radical surgery or radiotherapy. Carter et al. [53] recently demonstrated a significant association with disease upgrade in men being treated with AS with germline variants in *BRCA1/2/ATM,* with significantly more Gleason Grade Group (GGG) 1 upgrading to ≥ GGG3 compared with non-carriers (five-fold greater risk; adjusted HR 2.40, p = 0.046). (Fig. 4). This finding has significant implications for treatment decisions in men with known *BRCA2* or *ATM* germline variants diagnosed with localised PrCa.

San Francisco et al. [54] analysed predictors of progression in men with low-risk PrCa during AS (n = 120). They found men with a FH of PrCa (at least one FDR or second-degree relative) were more likely to experience disease progression than men without (HR 1.93, 95% CI 0.96, 3.90; p = 0.07) after a median follow-up of 2.4 years.

## Summary

There is now convincing evidence demonstrating a significantly increased risk of aggressive PrCa and poorer prognosis in men with a pathogenic germline variant in a DNA repair gene. Knowledge of a mans’ germline status therefore provides valuable information regarding prognosis, carries implications for offering targeted treatments and cascade testing for family members with respect to at least *BRCA1/2* variants. The inclusion of germline genetic testing for variants in genes such as *BRCA1/2, ATM* and *CHEK2* are likely to be incorporated into mainstream testing for men presenting with locally advanced or metastatic disease.

### Single nucleotide polymorphisms (SNPs) and polygenic risk scores (PRS)

Risk alleles occurring in ≥ 1% of the population are known as single-nucleotide polymorphism (SNPs). Prostate-cancer associated SNPs result in an elevated and potentially clinically relevant risk when multiple SNPs occur together, producing a cumulative effect as their risk is multiplicative (log additive). Increasing knowledge of polygenic disease heritability and susceptibility, and the ability to perform large GWAS of thousands of cases/controls and disease-specific SNP discovery allows us to construct risk scores based on an individual’s germline genetics (polygenic risk scores or ‘PRS’). The value of PRS emerged from the genotyping of thousands of individuals initially with common non-cancerous conditions (i.e. coronary artery disease) in order to investigate disease-specific genetic variants and their effects.

By measuring the genetic burden for a specific disease/trait, PRS provides a clinically useful tool in identifying groups of people at risk of a disease, for example to stratify men into a targeted screening regimens by only screening those at the greatest risk, ie those we can justify exposing to potential hazards of screening tests. The PRS is calculated as the sum of the weighted risk alleles, with the effect of each allele mapped from published GWAS.

### Germline single nucleotide polymorphisms (SNPs)

Large scale GWAS have led to the discovery of up to 170 SNPs specifically associated with PrCa risk [55–58]. Based on 147 SNPs in a meta-analysis by Schumacher et al. [59] approximately 28.4% of the familial risk in PrCa can be explained, with men in the top 1% of the risk profile having a 5.7-fold relative risk of developing PrCa compared with men in the 25–75th or ‘average’ centiles of risk (Table 2). Of note, the PRS effect increased with the presence of a FH or in those with a PrCa diagnosis ≤ 55 years. A risk model using a SNP profile with FH status could form part of a targeted screening strategy to those at highest risk, as discussed later in the PROFILE study.

**Table 2:** Reproduced from Schumacher et al. Estimation of PrCa risk by PRS using 147 risk SNPs. Men categorised into PRS percentiles based on the cumulative score distributed among controls

Risk category percentile	Relative risk	95% CI
< 1	0.15	0.11–0.2
1–10	0.35	0.32–0.37
10–25	0.54	0.51–0.57
25–75	1 (Baseline)	
75–90	1.74	1.67–1.82
90–99	2.69	2.55–2.82
≥ 99	5.71	5.04–6.48

Men in with a PRS in the highest percentile of risk (≥ 99%) have a RR of 5.71 compared to controls [59]

Reprinted by permission from Springer Nature: Nature Genetics. Association analyses of more than 140,000 men identify 63 new prostate cancer susceptibility loci, Schumacher et al. ©2018

Zheng & colleagues published their results examining the effect of the five commonest known SNPs associated with PrCa. They found their presence in combination with a FH accounted for 46% of the cases of PrCa in their cohort and conferred an odds ratio of 9.46 compared with men who had none of these factors, independent of PSA [58].

**Fig. 5:**
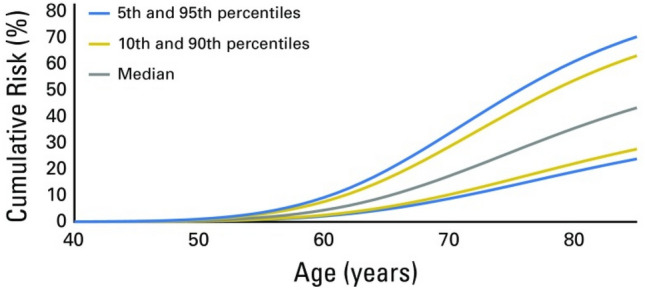
Reproduced from Lecarpentier et al. Predicted PrCa cumulative risk for male carriers of *BRCA2* variants by percentiles of PrCa polygenic risk score that was constructed by using results from population-based studies [60]. Reprinted with permission © 2017 American Society of Clinical Oncology. All rights reserved

Lecarpentier and colleagues investigated the use of SNP profiling as a means of predicting PrCa risk in 1802 men with *BRCA1/2* variants, based on 103 known PrCa susceptibility loci. They demonstrated an increasing PrCa risk for increasing PRS quartiles, with an estimated risk of (any) PrCa of 61% by age 80 in men with *BRCA2* variants who were in the 95^th^ percentile of risk according to their PRS*.* This study provides valuable information on the additional benefit of SNP profiling in this group of men for risk stratification, which ultimately has the power to inform the patient and clinician on timing and type of screening/intervention decisions [60]. These results indicate that a PRS could be informative in predicting individualised cancer risk for *BRCA1/2* variant carriers, a small but important group of men due to their high-risk status and could form the basis of an enhanced screening strategy for *BRCA1/2* variant carriers (Fig. 5). Until recently, no formal UK or international guidance exists regarding screening programmes for men with additional PrCa risks (such as *BRCA1/2* variant status or FH) were available but the EAU has very recently issued guidelines regarding screening for *BRCA2* germline variant carriers, recommending early PSA testing to men > 40 years old who carry a *BRCA2* germline variant [61].

## Prostate cancer screening

Screening for PrCa aims to detect clinically important cancers, whilst in parallel minimising men’s exposure to the morbidity of unnecessary prostate biopsies and diagnosing clinically insignificant PrCa. The US Preventive Services Taskforce (USPSTF) cited the benefits of PSA screening as ‘small and potentially none, and the harms are moderate to substantial’ [62]. The diagnostic accuracy and improved significant cancer detection rates resulting from the uptake of pre-biopsy MRI alongside a refined understanding of the influence of germline genetics and FH status on PrCa risk have led to Insights into how we can better risk-stratify men.

### FH analyses in ERSPC and PLCO trials

A subset analysis of European Randomised Screening Study of Prostate Cancer (ERSPC) (n = 4932) analysed the effect of FH in the Swiss cohort. Cumulative, screen-detected PrCa incidence over an 11 year period was significantly different between men with and without a FH (18% vs 12% respectively; HR 1.6). They reported FH along with age and baseline PSA as significant predictors of overall PrCa incidence, but only baseline PSA acted as an independent predictor for Gleason ≥ 7 cancer. When men were stratified by FH status, 5.1% of men with a FH of PrCa were found to have clinically significant cancer compared to 4% of men without a FH (no statistically significant difference) [63].

Examining the PLCO screening study data, Liss et al. found that when they specifically analysed all study participants with a FH, those who were screened had a trend towards decreased PrCa specific mortality and time to death, with a significantly higher incidence of PrCa and cancer-specific mortality in those with a FH compared to those without [64]. Abdel-Rahman analysed the relationship between PrCa incidence and a history of PrCa in FDR in 74,781 men from PLCO data. Similarly to ERSPC, a FH of PrCa was associated with a higher probability of cancer diagnosis (HR 1.59; 95% CI 1.48–1.70, P < 0.001) with the number of affected first-degree relatives correlating positively with risk. By FH status (one FDR with PrCa) across both study arms, 10.5% of men without a FH were found to have PrCa compared with 16.5% of men with a FH. There was no statistically significant difference in tumour stage, histology, PSA or patient age between cancer cases in men with and without a FH. When analysing by screening arm vs non-screening arm, FH in a FDR and the number of FDRs was significantly associated with PrCa mortality (HR 1.89; 95% CI 1.15–3.10, p = 0.012) in the non-screening arm compared to the interventional arm [65] suggesting a benefit to screening this group.

### Family history analyses in the placebo arms of the PCPT and REDUCE trials

The Prostate Cancer Prevention Trial (PCPT) investigated the use of Finasteride, a 5-alpha-reductase-inhibitor (5ARI) in PrCa prevention. In the placebo arm of the study, men either underwent end of study biopsy (at 7 years) or a clinically-mandated biopsy if PSA was ≥ 4.0 ng/ml or abnormal DRE at any of the men’s annual study visits up to year 7. Of the 4,692 men in the placebo arm who underwent evaluation, 1,147 cancers were detected (24%). Of those available for evaluation, 237 were Gleason 7, 8, 9 or 10 (22%) [66]. In a separate analysis of 5,519 men in the placebo arm of this study, men with a FH (16% of the cohort) of PrCa had an odds ratio (OR) of 1.31 (95% CI 1.1–1.5) for harbouring PrCa on any form of prostate biopsy throughout study follow-up. The median PSA of this cohort at study entry was 1.5 ng/ml with 88% of men having a PSA ≤ 4.0 ng/ml. Approximately 24% of men with a FH who underwent prostate biopsy had (any grade) PrCa compared with 17% of men without a FH. FH was not associated independently with high-grade disease. Approximately 95% of this cohort was of European origin [67].

The REDUCE study was a 4-year RCT comparing efficacy of Dutasteride compared to placebo in preventing the development of PrCa in men defined at the study entry as being at an increased risk for PrCa (due to abnormal PSA/DRE). A sub-analysis of the study also examined the effect of FH on PrCa incidence at time of biopsy in both treatment and placebo arms. In the placebo arm, they found PrCa (all grades) in 23% of men undergoing biopsy with a FH compared to those without (19%) in the placebo arm, and found a 31% risk reduction (RR) in PrCa with Dutasteride [68, 69].

## Investigating the role of targeted screening in men with a genetic predisposition

We know men with a FH have an elevated risk of an early onset of the disease and men with inherited germline variants in DNA repair genes are particularly at risk for harbouring aggressive histology. It is therefore sensible to investigate the feasibility and efficacy of targeted screening programmes in these important groups of high-risk men, who are well placed to truly benefit from early disease detection and treatment.

### Genetic scores and prostate cancer screening

There is evidence to suggest genetic based scores improve PrCa detection and risk stratification. Using 14 known PrCa associated SNPs and the presence/absence of a FH of PrCa, Xu et al. reported an OR of 4.92 for developing PrCa for men with a positive FH and ≥ 14 risk alleles [70]. Using data from the REDUCE trial, which assessed the chemopreventative benefits of Dutasteride, Kader and colleagues analysed germline DNA from 1654 controls. These men all had an initial negative prostate biopsy, with subsequent prostate biopsies at 2 and 4 years. They found adding a genetic score based on 33 risk SNPs with clinical variables was an independent predictor for PrCa on repeat prostate biopsy, and demonstrated the ability to reduce the number of repeat biopsies required [71]. Recently, Na et al. investigated the association between a genetic risk score (GRS) and patient age at PrCa diagnosis compared to the association with FH. They performed a cohort study of 3225 white men (also from the REDUCE trial), and constructed a GRS based on 110 known PrCa risk SNPs for each participant. They found higher GRSs were associated with earlier age at PrCa diagnosis, independent of FH status [72].

Callender et al. investigated the cost-effectiveness and benefits/harms of using a PRS tailored screening program by way of a simulated model. They compared three screening models; no screening, age-based screening (PSA every 4 years from age 55 to 69) and risk-tailored screening (PSA every 4 years only in men whose risk is at or above a certain absolute risk threshold based on their PRS). They compared cost, overdiagnosed cancers and amount of PrCa-related deaths averted due to screening between models. They found an age-based program prevented the most deaths but caused a greater amount of overdiagnosed cancers whereas a precision-based screening strategy averted a third more cases of overdiagnosis but averted fewer PrCa-specific deaths than the age-based model [73].

Pashayan et al. assessed the implications of using polygenic risk scoring (PRS) on reducing over-diagnosis. They constructed a PRS on 17,000 men aged 50–69 from three large studies (ProtecT, SEARCH and UKGPCS) using 66 known PrCa risk SNPs, separating men with and without PrCa into risk quartiles. By using this method, they derived probabilities of overdiagnosis per risk quartile. They estimated from lowest risk quartile to the highest, a proportion of 43, 30, 25 and 19% of cancers were ‘overdiagnosed’ with the rate of overdiagnosis decreasing with increasing polygenic risk. They estimated a 56% reduction in over-diagnosis between the lowest risk quartile and the highest [74] suggesting a PRS could be used to risk-stratify men in higher risk categories who would benefit the most from screening and reducing harms of overdiagnosis.

### Germline genetic testing guidelines

Only recently has published guidance emerged on advising clinicians when to perform germline testing in men with PrCa and in which specific groups.

#### Philadelphia prostate cancer consensus conference

The Philadelphia Prostate Cancer Consensus Conference (PPCCC), ‘Role of Genetic Testing for Inherited Prostate Cancer Risk’ was held in 2017 [75] and was the first comprehensive, multidisciplinary meeting to address a genetic evaluation framework for HPC. This meeting’s final recommendations emphasised future research should focus on developing a working definition of familial PrCa for clinical genetic testing and exploring the use of genetic tests for PrCa management.

The second PPCCC in 2019, ‘Implementation of Germline Genetic Testing for Prostate Cancer’ [76] provides an up to date, multi-disciplinary progressive framework for guiding clinicians. Germline panel testing (*BRCA1/2, MMR* and *ATM*) and somatic tumour testing were recommended for all men with mPrCa and men with suspected HPC. Other FH criteria for panel testing included: men with one FDR with PrCa, men with two or more male relatives with one of the following: PrCa < 60 years old, death from PrCa (any age), mPrCa (any age). Consideration of germline panel testing should be considered in men with non-metastatic but high-grade (≥ GGG4), ≥ T3a, intra-ductal pathology or Ashkenazi Jewish heritage. Screening is advised for men with a known *BRCA2* variant to begin aged 40 or 10 years prior to the youngest PrCa diagnosed in the family. No specific new advice for screening or genetic testing was present for black men due to lack of available additional genetic data in this group.

#### National comprehensive cancer network (NCCN)

The 2020 NCCN ‘Clinical Practice Guideline in Oncology: Genetic/Familial High-Risk Assessment: Breast, Ovarian and Pancreatic; Version 1.2021′ state *BRCA1/2* (and other cancer susceptibility genes) testing is clinically indicated in men (any age) with a personal history of metastatic or intraductal PrCa. Testing is also indicated in men with a personal history of Gleason ≥ 7 with: Ashkenazi Jewish ancestry, ≥ 1 close blood relative (first, second or third-degree relative on same side of family) with breast cancer < 50 years of age/ ≥ 1 close blood relative with ovarian/pancreatic/metastatic PrCa/intraductal PrCa at any age/ ≥ 2 close relatives with breast or PrCa (any grade) at any age. Unaffected men with a FH of PrCa with a first or second-degree blood relative meeting any one of the previously mentioned criteria would also qualify for germline testing [77].

The latest (2020) NCCN Clinical Practice Guidelines in Oncology: Prostate Cancer Version 3.2020 also recommend germline genetic testing for men with NCCN high-risk, very high risk regional or metastatic PrCa, for all men of Ashkenazi Jewish ancestry, a known FH of *BRCA1/2* or Lynch Syndrome, a FH of brother/father/multiple family members diagnosed with PrCa (except GGG1) at < 60 years old OR who died from PrCa. Testing is also advised for men with a FH of $\ge$ 3 cancers on the same side of the family including bile duct, breast, ovary, colorectal, endometrial, pancreatic, kidney, melanoma, small bowel, urothelial or prostate (except GG1) [78].

The latest (2020) NCCN Clinical Practice Guidelines in Oncology: Prostate Cancer Early Detection Version 2.2020 August 24, 2020 recommends annual PSA screening for men with known *BRCA1/2* pathogenic variants due to their increased risk of subclinical, high-grade disease, increased mortality and earlier age at diagnosis [79].

#### ESMO 2020

Recently published guidance from ESMO ‘Clinical Practice Guidelines for diagnosis, treatment and follow-up’ also recommends germline testing for *BRCA2* and other DNA repair genes in all men with advanced/metastaticPrCa, regardless of tumour features or FH status, and in all men diagnosed with PrCa with a FH of cancer (at least two close relatives on the same side of the family) linked to hereditary cancer syndromes (ie breast, ovarian, pancreatic, prostate). A recommendation for the testing of tumour tissue for homologous recombination genes and *MMR* defects (or microsatellite instability) in men with mCRPC is also made [80].

### Future directions

It is unclear at present how PRS relates to the probability of detecting existing PrCa in asymptomatic men with a FH, many of whom will have low PSAs. The predictive value of SNP profiling in men presenting with a PSA of 1–3 ng/ml was assessed by Nordstrom et al. [23], who found that a risk score based on 49 SNPs was a significant predictor of a positive biopsy (p = 0.028). Based on current clinical practice if these men were following a PSA screening protocol, they would not fulfil clinical criteria for urological referral. In the PROFILE feasibility study, the predictive value of a PRS for men with a FH was analysed. No significant association between the PRS and PrCa diagnosis was found in 100 healthy men with a FH of PrCa undergoing screening prostate biopsy irrespective of PSA. However, the number of cancers diagnosed in this group of men (mean age 53) with a low median PSA (1.3) was sizeable (25% had PrCa found on screening biopsy of whom 48% had clinically significant disease). Twelve men with Pr Ca had a PSA < 3 (52%). No adverse psychosocial variables were noted. However it was not designed to be powered to answer this query and was only undertaken to see if such an approach was acceptable [81].

Presently, the full PROFILE study (NCT02543905) is recruiting 350 men with a FH of PrCa and 350 men of African ancestry, investigating the role of targeted screening in men with a genetic susceptibility to PrCa. Germline genetic analysis of 130 SNPs will be correlated with outcome at upfront prostate biopsy (regardless of PSA) at study entry in men aged 40–69. This prospective, targeted screening study will determine the association of genetic profiling with prostate biopsy result in those with a genetic susceptibility to PrCa undergoing targeted screening. PrCa incidence, aggressiveness and incidence of abnormal pre-biopsy MRI and its value in this cohort will also be assessed.

Currently, the IMPACT study (NCT00261456) has enrolled over 3,000 men (variant carriers and controls) across multiple countries to investigate the outcomes of targeted PSA screening in men with *BRCA1/2* and MMR (*MSH2, MSH6, MLH1*) germline variants with annual PSA and a biopsy threshold of 3.0 ng/ml. Early results in the *BRCA1/2* cohort have suggested a screening strategy in this population is beneficial for men with a *BRCA2* variant, with variant carriers having with a higher rate of PrCa diagnosis, at a younger age and having more significant disease than non-carriers [82]. Interim results for the Lynch Syndrome cohort are awaited.

Mano et al. have published their results of prospectively screening 196 Israeli male *BRCA1/2* variant carriers (aged > 40) for five cancers including PrCa. The rate of PrCa in *BRCA1* variant carriers (8.6%) was twice that of *BRCA2* variant carriers (3.8%), screening all men using annual PSA and DRE (neither PSA screening threshold or cancer characteristics reported) [83]. Within in the same institution, Golan et al. reported on 138 men referred to their Risk Clinic for germline genetic testing due to a FH of PrCa, a FH of multiple other malignancies or a known germline variant. Men with a FH of PrCa comprised 64% of their cohort, and 25% had a known germline variant. A total of 18% were found to carry a germline variant in *BRCA1/2*, *CHEK2*, *HPC2*, *ATM*, *MLH1*, *MSH2* or *MSH6*. This cohort is likely to be enriched for variants due to Jewish ethnicity [84]. Das et al. have also reported their intention to study a prospective cohort of men with known germline variants, managed in a high-risk clinic [85]. Their ‘High-Risk’ clinic will utilise PSA, DRE, SelectMDx™ and MRI in a risk-algorithm.

The ‘Genetic Testing for Men With Metastatic Prostate Cancer’ (GENTleMEN) study is a prospective, observational study run by the University of Washington (NCT03503097), currently recruiting 2,000 men with metastatic PrCa to undergo germline genetic analysis (participants will provide a postal saliva) and provide patient-reported-outcome-measures associated with genetic testing. Participants receive web-based or paper questionnaires and saliva collection kits via mail or in person. Participants then receive phone-based genetic counseling if they are identified to have an inherited variant in *BRCA1/2, ATM* and other genes [86].

The STOCKHOLM3 study (STHLM3) [87], reported in 2015, was the first population based PrCa screening study that prospectively assessed a targeted screening approach. The study used a screening model combining liquid biomarkers (including PSA), 232 risk SNPs and clinical variables (e.g. age, FH) and compared this with PSA alone (using a threshold of ≥ 3 ng/ml) [87]. They reported the sensitivity for the detection of clinically significant risk PrCa was improved with the STHLM3 model (AUC 0.74 vs 0.56) compared to PSA and also reduced the number of biopsies by 32% and avoided 44% of benign biopsies. Taking this approach further, the STHLM3-MRI project aims to improve the PrCa diagnostic pathway by investigating the role of the STHLM3 test as a triage tool to asses non-inferiority to a standard diagnostic pathway using PSA and standard systematic biopsy. The pathway will randomise men at the point of diagnostic test after either a PSA ≥ 3 ng/ml or STHLM3 > 11, with diagnostic test either being a traditional systematic or MRI-guided biopsy [88]. The ReIMAGINE Prostate Cancer Screening study (NCT04063566) is currently inviting PSA naive men in the general population aged 50–75 via their GP to undergo prostate MRI. Those with MRI lesions assigned a PIRADS score of ≥ 3 (or with a PSA density > 0.12) will be referred for standard further PrCa diagnostic tests. This study will evaluate the feasibility of using prostate MRI as a population screening tool and the prevalence of MRI-detected PrCa across a spectrum of PSAs.

BARCODE1 will be the first prospective UK study to utilise a germline 130 SNP profile to target PrCa screening in the general population, recruiting patients via their general practitioners (GPs). Intervention (based on a PRS falling in the top 10% of risk) in BARCODE1 is in the form of an MRI guided prostate biopsy in those in the top 10% of the PRS. With the increasing interest in use of MRI as a triage tool to decide whether men presenting with symptoms or a raised PSA can safely avoid a biopsy, BARCODE1 will allow an assessment of the utility of MRI in men who have an increased genetic risk of prostate cancer based on a PRS. In the BARCODE1 pilot study, uptake of SNP profiling by providing a saliva sample via GPs was 26% with 25/303 identified for intervention based on a PRS falling in the top 10% of risk; 45% of these men had an abnormal MRI with (any) cancer detected in 38.8% [89].

## Conclusion

We are now in a position to translate our understanding of the polygenic nature of PrCa risk to informing and improving screening strategies, by stratifying men into risk categories based on their genetic and FH status and undertaking screening research studies. The accuracy of PrCa diagnostics, headlined by the PROMIS and PRECISION trials [90, 91] has been revolutionised by pre-biopsy MRI, improving cancer detection by targeting sampling to areas of abnormality in place of systematic TRUS biopsies, ultimately reducing rates of overdiagnosis. The aforementioned prospectively performed IMPACT, PROFILE and BARCODE1 studies will give practical insight into the role of genetic-based screening in PrCa detection in high risk men and the ability of a targeted strategy to divert ‘low risk’ men from invasive diagnostics tests and funnel ‘high-risk’ men towards the most accurate test, whilst in parallel minimising the risk of overdiagnosis. The next decade will see further translational research into applying knowledge of germline genetics and incorporating men’s FH status into truly personalised PrCa screening, diagnostics and treatment.

## Data Availability

All data resulting from literature search available on Pubmed.
